# Extramedullary hematopoiesis presenting as a compressive cord and cerebral lesion in a patient without a significant hematologic disorder: a case report

**DOI:** 10.1186/1752-1947-4-319

**Published:** 2010-10-12

**Authors:** Amir Saied Seddighi, Afsoun Seddighi

**Affiliations:** 1 Shohada Tajrish Hospital, Beheshti University of Medical Sciences, Tehran, Iran; 2 Rajaie Hospital, Qazvin University of Medical Sciences, Qazvin, Iran; 3 Neurofunctional Research Center, Shohada Tajrish Hospital, Beheshti University of Medical Sciences, Tehran, Iran

## Abstract

**Introduction:**

Intracranial or spinal compressive lesions due to extramedullary hematopoiesis have been reported in the medical literature. Most of the reported cases are extradural lesions or, on rare occasions, foci within another neoplasm such as hemangioblastoma, meningioma or pilocytic astrocytoma. Often these cases occur in patients with an underlying hematological disorder such as acute myelogenic leukemia, myelofibrosis, or other myelodysplastic syndromes. Such lesions have also been reported in thalassemia major.

**Case presentation:**

We report the case of a 43-year-old Iranian woman in whom extramedullary hematopoiesis presented as a compressive cord lesion and then later as an intracranial lesion.

**Conclusions:**

To the best of our knowledge, we document the first reported case of sacral, lumbar, thoracic and cranial involvement in the same patient with extramedullary hematopoiesis, which seems both rare and remarkable.

## Introduction

Intracranial or spinal involvement, manifesting as epidural lesions, due to extramedullary hematopoiesis (EMH) is rare. The intracranial lesions are also reported as foci within another intracranial neoplasm such as hemangioblastoma, meningioma or pilocytic astrocytoma [1,2]. Extramedullary hematoopoiesis usually occurs in patients with a significant hematologic disorder like acute myelogenic leukemia (AML), myelofibrosis (MF), myelodysplastic syndromes or thalassemia major [1-3]. Prior to our case report and to the best of our knowledge, there has been no report of such a problem presenting as compressive spinal and cranial lesions in the same patient without a significant hematologic problem. The low incidence of EMH in central nervous system indicates that cells with hematopoietic potential find little supporting environment in CNS [3].

## Case presentation

**Figure 1:**
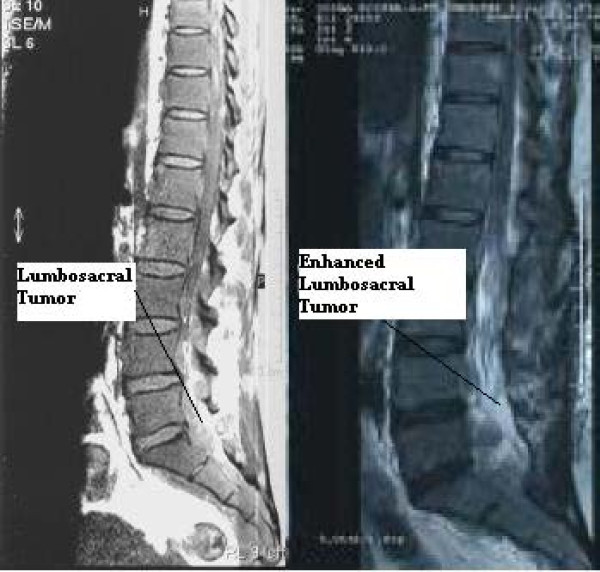
**An MRI of the lumbosacral spine (left) showed an extradural lesion extending from L3 to S3 (left), which became enhanced after a contrast injection (right)**.

**Figure 2:**
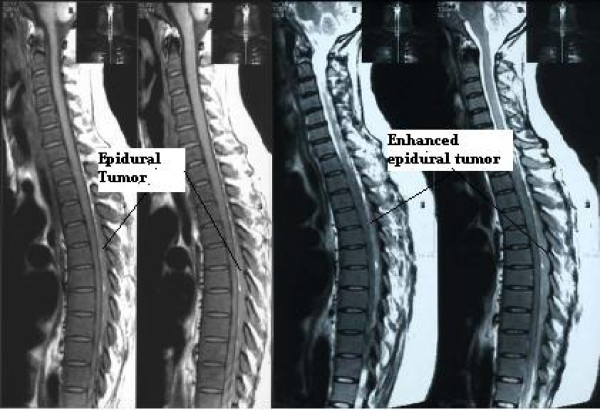
**An MRI of the thoracic spine showed an extradural lesion extending from T3 to T11 (left) which became enhanced after a contrast injection (right)**.

Our case report, a 43-year-old Iranian woman, came to us complaining of back pain radiating to both of her lower extremities which had started two months previously. The pain was non-responsive to conventional medical treatments. In her general physical exam, mild splenomegaly was noted. A neuroexam showed decreased strength in both of her distal lower extremities (motor strength = 4/5) and absent deep tendon reflexes in her lower extremities. She had a limping gait due to pain. A sensory exam indicated problems in the L4 and L5 dermatomes. Her sphincter function was normal. A lumbosacral magnetic resonance imaging (MRI) scan showed an abnormal para-vertebral mass extending from L3 to S3 which became enhanced after a contrast injection (Figure 1). In a dorsal spine MRI, another epidural lesion, extending from T3 to T11 with mild compression over the thoracic cord, was identified which became enhanced after a contrast injection (Figure 2). An MRI of both her cervical spine and brain were normal. An abdominal and pelvic computed tomography (CT) scan with and without intravenous and oral contrast media showed no abnormality. Para-clinical data showed an elevated erythrocyte count, decreased mean corpuscular volume (MCV), mean corpuscular hemoglobin (MCH) and mean corpuscular hemoglobin concentration (MCHC). Her retic count was also elevated. Her white blood cell count showed mild leukocytosis and a few myelocytes and band cells.

Her red blood cell count was 7.66 × 10^6^/mm^3^; her haemoglobin level was 20.5^g^/dl; her hematocrit level was 67.3%; her mean corpuscular volume was 67 fl; her mean corpuscular hemoglobin level was 17.6 pg; her mean corpuscular hemoglobin concentration was 26.3^g^/dl; her reticulocyte level was 3.5%; her platelet count was 24,6000/mm^3^; her white blood cell count was 14.6 × 10^3^/mm^3^; the percentage of polmorphonuclear cells in her blood was 46%; the percentage of monocytes was 2%; the percentage of band cells was 1%; the percentage of lymphocytes was 48%; and the percentage of myelocytes was 3%.

**Figure 3:**
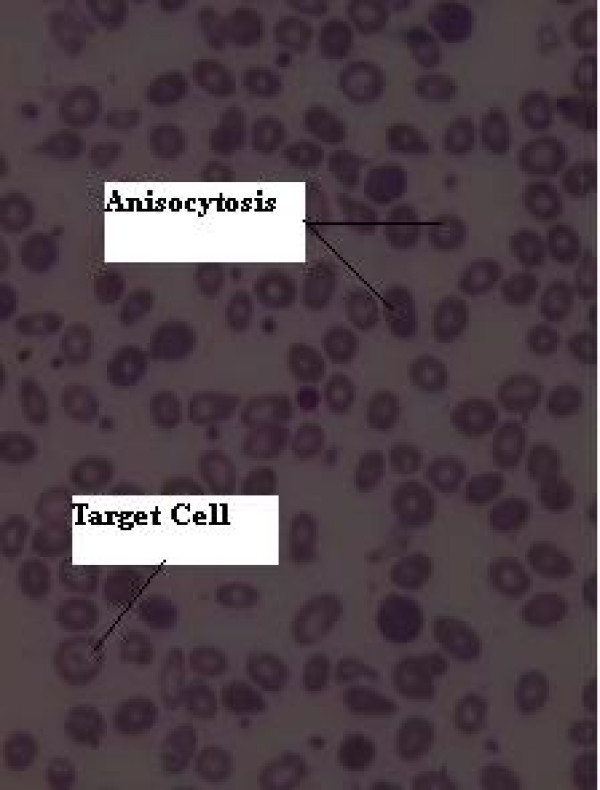
**A peripheral blood smear showed nucleated-RBC, anisocytosis, microcytosis, hypochromia, poikilocytosis, tear-drop spherocytes and target cells**.

A consultation with the hematologist and a review of the peripheral blood smear yielded nucleated-RBC, anisocytosis (1+), microcytosis (2+), hypochromia (2+), poikilocytosis (2+), tear drop spherocytes and target cells (Figure 3). Her Hb electrophoresis was within normal range. Her coagulation profile was intact. She had no related positive family history. She did not smoke at all. A bone marrow aspiration revealed normal appearing megakaryocytes and normoblasts with shrinked cytoplasm in the late stages of development in erythroid order and a mild shift to the left and increased lymphocyte count in white order all indicating an increase hematopoietic activity. Her hemoglobin electrophoresis was normal so a diagnosis of thalassemia was ruled out. Her serum erythropoietin level was 11 mU/ml, which was normal. An arterial blood gas analysis showed normal oxygen saturation and no hypoxia.

**Figure 4:**
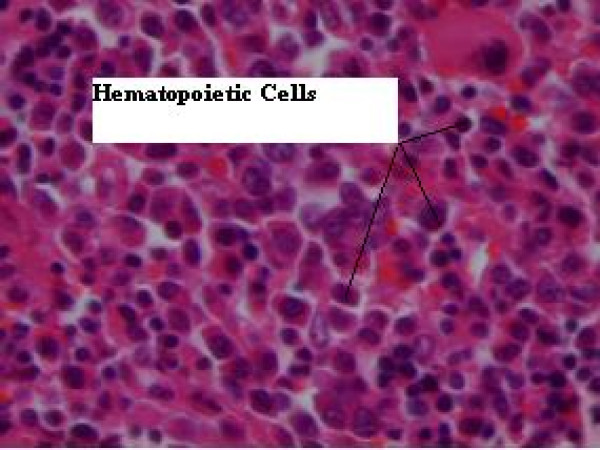
**A histopathologic view of the resected epidural lesion, showing considerable replacement of epidural fat with hematopoietic cells predominantly from erythroid and granulocytic clones and also scattered megakaryocytes**.

A two-week steroid therapy was of no benefit and her neurologic status deteriorated, so we planned to perform a surgical decompression. Since she manifested dominantly with lower motor neuron problems and the dorsal lesion was very extensive with a mild compressive effect over the cord, we decided to decompress the lumbar area. She was positioned prone and a bilateral laminectomy from L2 to S1 was performed. The extensive dark-grayish epidural lesion had a fragile consistency. We removed as much of the lesion as possible. The thecal sac and the roots were successfully decompressed. Microscopic studies showed considerable replacement of epidural fat with hematopoietic cells, predominantly from erythroid and granulocytic clones and also scattered megakaryocytes (Figure 4).

The adjuvant treatment continued with whole-spine radiotherapy, which was accompanied by her gradual recovery from paraparesis. Follow-up contrast studies undertaken every six months showed no evidence of any recurrence.

Twenty-one months after her first visit, she came back complaining of headaches and visual blurring beginning two weeks previously. A clinical assessment revealed a grade 4 papilledema and mild paraparesis (muscle strength = 4/5) associated with generalized hyper-reflexia and extensor plantar reflexes.

**Figure 5:**
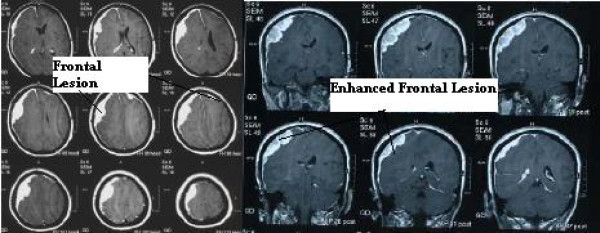
**A brain MRI of the patient performed during her second admission showed an extradural lesion in the right frontoparietal region and a smaller lesion in the left frontal region (left) which became enhanced after a contrast injection (right)**.

A whole axis contrast-enhanced magnetic study revealed a large extra-axial mass in her right frontal area, causing more than 10 mm midline shift, and a smaller mass in her left frontal area. The lesion became enhanced after a contrast injection and showed a dural tail sign. Patchy-enhancing foci was also evident in the meninges (Figure 5). A spine study was negative except for post-operative and post-radiation changes. A para-clinical study only showed mild thrombocytopenia and mild leukocytosis.

Her red blood cell count was 4.76 × 10^6^/mm^3^; her hemoglobin level was 11.9/dl; her hematocrit level was 37.6%; her mean corpuscular volume was 79 fl; her mean corpuscular hemoglobin level was 23 pg; her mean corpuscular hemoglobin concentration was 29^g^/dl; her platelet count was 78 × 10^3^/mm^3^; her white blood cell count was 32.4 × 10^3^/mm^3^; the percentage of polymorphonuclear cells in her blood was 71%; the percentage of lymphocytes was 13%; the percentage of monocytes was 5%; the percentage of myelocytes was 1%; the percentage of stab cells was 9%; and the percentage of metamyelocytes was 1%.

She underwent a platelet transfusion because of her thrombocytopenic state. According to consultation with the hematologist, her thrombocytopenia was explained by the hypersplenism.

A course of steroid therapy was performed but it was unsuccessful. She showed signs of increased intracranial pressure, so we decided to proceed with surgery.

To reduce the intracranial pressure, a right frontoparietal craniotomy and resection of the larger lesion was performed, which was accompanied with duraplasty to improve her clinical condition. During the operation, the mass appeared dark and vascular just below the dura mater and adhering to, and in some places invading, the pia matter. Immediately after the operation, she became hemiparetic on her left side and a CT scan showed severe edema compressing her right ventricle and no evidence of hemorrhage. The medical treatment for edema was initiated but in the second post-operative day, she became hemiplegic on her left side. The hemiplegia gradually and slowly improved, although modestly. A microscopic study of the lesion indicated erythroid, myeloid and megakaryocytic proliferation indicating EMH. After consultation with hematologists and oncologists, she was sent for cranial radiation. In her follow-up visit, six months after admission, there was no evidence of any radiologic recurrence of the supratentorial lesion and the patient's condition had improved. The follow-up visits continued every six months and after two years there had been no evidence of any radiologic or clinical recurrence and the patient's condition had improved.

## Discussion

Involvement of the neuroaxis due to extramedullary hematopoiesis is not common. The reported cases occurred due to some major hematologic disorders such as myelodysplastic syndromes, acute myelogenic leukemia or thalassemia major [1,2]. In very rare cases, these lesions have also been seen mixed with some neoplasms including meningioma, pilocytic astrocytoma or hemangioblastoma [1-3]. The low incidence of EMH in CNS indicates that cells with hematopoietic potential find little supporting environment in CNS [3]. To the best of our knowledge, we document the first reported case of sacral, lumbar, thoracic and cranial involvement in the same patient, which seems remarkable. Involvement of neuraxis in EMH is rare and most reported cases are intracranial [1-9].

Suggested treatments for these lesions include surgical removal and a combination of chemo-and radiotherapy in cases with hematologic malignancies, and blood transfusion when the underlying cause is thalassemia major, myelofibrosis or myelodysplastic syndromes.

At the first presentation of our case report, her hemoglobin level was high. She had no prior history of smoking. Her erythropoietin level and arterial blood gas analysis were normal. A bone marrow biopsy did not indicate myelofibrosis or a solid cancer metastasis and she has been disease free for two years, so these diagnoses were ruled out. We did not find the cause of her high hemoglobin level; however, it was not seen in the follow-up laboratory tests.

In our experience, she showed no sign of anemia to justify transfusion and surgical resection was considered necessary on both occasions due to the significant compressive effect and her deteriorating condition. Radiation as a treatment modality caused the thoracic lesion to disappear and there has been no evidence of any recurrence during follow-up checks. An increase of cerebral edema after surgical resection of these lesions has been reported by Gregorios *et al. *although EMH has occurred in a malignant meningioma in his report [8].

## Conclusions

Although CNS involvement in EMH is very rare, this entity deserves attention as a differential diagnosis in patients with an underlying hematologic disorder.

## Abbreviations

CT scan: computed tomography scan; EMH: extramedullary hematopoiesis; HCT: hematocrit; Hgb: hemoglobin; MCH: mean corpuscular hemoglobin; MCHC: mean corpuscular hemoglobin concentration; MRI: magnetic resonance imaging; Plt: platelet; RBC: red blood cell; U: unit; WBC: white blood cell.

## Consent

Written informed consent was obtained from the patient for publication of this case report and any accompanying images. A copy of the written consent is available for review by the Editor-in-Chief of this journal.

## Competing interests

The authors declare that they have no competing interests.

## Authors' contributions

ASS analyzed and interpreted the patient data regarding the lateral sacral meningocele and performed the surgery. AS performed the review of literature and was a major contributor in writing the manuscript. All authors read and approved the final manuscript.
